# Research Knowledge Translation in Sensory Integration-Based Therapy: Exploring Subjectivity of Clinical Expertise

**DOI:** 10.1177/00084174231223875

**Published:** 2024-01-17

**Authors:** Diane Elizabeth Bird, Tanya Rihtman

**Keywords:** Clinical expertise, Evidence-based occupational therapy, Qualitative research, Sensory integration, Translational science, Ergothérapie fondée sur les données probantes, Expertise clinique, Intégration sensorielle, Recherche qualitative, Recherche translationnelle

## Abstract

**Background.** Clinical expertise is the mechanism through which practitioners implement other components of evidence-based practice (EBP). Within occupational therapy practice, intervention approaches that are both closely and loosely aligned with Ayres’ Theory of Sensory Integration are widespread, offering a unique opportunity to investigate the subjective nature of clinical expertise in EBP. **Purpose.** This qualitative study explored motivations to offer sensory integration-based interventions, and factors informing occupational therapists’ clinical decision making in relation to an arguably contentious evidence base. **Method.** Six post-graduate sensory integration trained UK occupational therapists participated in individual semi-structured interviews. Interviews were transcribed, member-checked and analyzed using thematic coding analysis. **Findings.** Despite sound understanding of theory and continuous efforts to develop clinical knowledge, non-traditional hierarchies of evidence notably inform clinical decisions. The clinical expertise required for integration of patient preferences, clinical state and circumstances, and research evidence is informed by pragmatic responses to facilitators and barriers across contexts, combined with unique profession-specific identity factors. **Implications.** While empirical healthcare research is ideally undertaken under controlled conditions, realities of clinical practice are rarely so clear cut. Study findings highlight important subjective factors that are central to real-world research knowledge translation and further understanding of the clinical expertise component of EBP.

## Abrégé

**Description.** L’expertise clinique est le mécanisme par lequel les praticiens mettent en œuvre d’autres composantes de la pratique fondée sur des données probantes (PFDP). Dans la pratique de l’ergothérapie, les approches d’intervention liées de près et de loin à la théorie de l’intégration sensorielle d’Ayres sont très répandues, offrant une occasion unique d’étudier la nature subjective de l’expertise clinique dans la PFDP. **But.** Cette étude qualitative explore les motivations derrière l’offre d’interventions basées sur l’intégration sensorielle, et les facteurs influençant la prise de décision clinique des ergothérapeutes par rapport à une base de données probantes controversée. **Méthodologie.** Six ergothérapeutes britanniques formés à l’intégration sensorielle aux cycles supérieurs ont participé à des entretiens individuels semi-structurés. Les entretiens ont été transcrits, vérifiés par les membres, et ont fait l’objet d’une analyse thématique. **Résultats.** Malgré une bonne compréhension de la théorie et des efforts continus pour développer les connaissances cliniques, des hiérarchisations non traditionnelles des données probantes influencent de matière notable les décisions cliniques. L’expertise clinique nécessaire à l’intégration des préférences du patient, de l’état et des circonstances cliniques ainsi que des données issues de la recherche est éclairée par des réponses pragmatiques aux facilitateurs et aux obstacles dans tous les contextes, combinées à des facteurs identitaires uniques et particuliers à la profession. **Conséquences.** Alors que la recherche empirique sur les soins de santé est idéalement menée dans des conditions contrôlées, les réalités de la pratique clinique sont rarement aussi tranchées. Les résultats de l’étude mettent en évidence des facteurs subjectifs importants qui jouent un rôle central dans l’application des connaissances issues de la recherche dans le monde réel et sont essentiels à une meilleure compréhension de la composante « expertise clinique » de la PFDP.

## Introduction

The initial conceptualisation of evidence-based practice (EBP) comprised three overlapping, equally important components: clinical expertise, patient preference and research evidence (Sackett et al., 1996). This conceptualisation has subsequently evolved, with the three central EBP components updated (clinical state and circumstance, patients’ preferences and actions, and research evidence) while clinical expertise has shifted to become the mechanism through which the individual practitioner implements the central ingredients of EBP (Haynes et al., 2002). Haynes et al. (2002) define clinical expertise as, “the general basic skills of clinical practice as well as the experience of the individual practitioner” (p. 37).

This definition, together with the relocation of clinical expertise as that which facilitates integration of the other EBP components, introduces an inherent subjectivity within EBP. While initially introduced from a more medically-focused paradigm, recent decades have brought about profession-specific exploration of EBP. Within occupational therapy practice, the professional requirement for EBP is unquestionable and practice guidelines highlight the role of professional expertise and expert opinion in clinical decision making (World Federation of Occupational Therapists, 2021). Research into occupational therapists’ EBP implementation (Krueger et al., 2020; Samuelsson & Wressle, 2015) has highlighted a need to further explore the subjectivity of clinical decision making processes when occupational therapists implement EBP. There remains a lack of understanding of the influence of subjective, individual personal clinical experiences, which combine with practitioner use of different types of clinical reasoning (e.g., scientific, pragmatic, conditional etc. [Unsworth, 2017]), coupled with adherence to the centrality of holism as a long-established, core professional value (where “the individual is viewed as an integrated, balanced being, with the capacity to determine what is needed for health”) (McColl, 1994, p. 74), to inform the use of clinical expertise within EBP.

Integration of new knowledge into action requires, by definition, a change of behaviour on the part of the clinician. Cane et al. (2012) describe numerous challenges in applying theories of human behaviour change to the design of implementation science research, and claim that these challenges of application lead to difficulties in identifying specific processes underlying successful behaviour change. To address this, they propose the Theoretical Domains Framework (TDF) as an integrative framework of behaviour change theories, which aims to “simplify and integrate a plethora of behaviour change theories and make theory more accessible to, and usable by, other disciplines” (Cane et al., 2012, p. 2). Considering the central role of clinical expertise in EBP, the TDF may be useful in supporting our understanding of factors outside of research evidence in health care provider behaviour change and clinical decision-making (Cane et al., 2012).

In addition to theories exploring behavioural change of clinicians (Cane et al., 2012), implementation of research evidence for occupational therapy practice, as part of an EBP process, is dependent on human processes of knowledge translation (Straus et al., 2009). Paez (2018) notes that the incorporation of evidence with patient values and circumstances should occur “in order to fulfil the practitioner's ethical obligation to best serve the patient and do not harm. Expertise plays an important role in that process” (p. 223). Furthermore, there is growing understanding of the need to move away from standard single-hierarchy research-evidence models (Tomlin & Borgetto, 2011), with increasing recognition that Western biases underpin such hierarchical organisation of scientific knowledge (Nagendra, 2018).

One area of clinical occupational therapy practice which could potentially provide useful insight into this central—yet subjective—component of EBP is that of sensory integration (SI) and SI-based occupational therapy. The scientific methods used by A Jean Ayres in the 1970s to develop SI theory marked the first attempt within the occupational therapy profession to present an evidence-based approach linking theory to clinical application (Mailloux & Miller-Kuhaneck, 2014). The primary premise of Ayres’ theory was that observable human behaviour reflects underlying neurological function (Reynolds et al., 2017) and that deficits in an individual's ability to integrate and use sensory information in an adaptive manner, result in participation limitations (Smith Roley et al., 2007).

Since its introduction in the 1970s, the application and implementation of SI theory has been used and interpreted in different ways, leading to a variety of SI-based (Brown et al., 2019) approaches founded on principles of SI but which do not fit within the purview of SI treatment as originally intended by Ayres (Reynolds et al., 2017). To tackle the resultant research and evidence confusion that appears to have stemmed from semantic debates coupled with inherently subjectivity clinical application, the term Ayres Sensory Integration (ASI®) was ultimately trademarked (Smith Roley et al., 2007). However, despite these attempts to ensure clarity, “contention” (Pollock, 2009, p. 9) has been described regarding SI-based interventions, with their efficacy, cost-effectiveness and evidence base all debated within occupational therapy communities (American Occupational Therapy Association, 2015; Novak & Honan, 2019) and by other professionals (Barton et al., 2015).

Regardless of the strength of academic feeling, “sensory therapy” remains common in the real world of occupational therapy practice. For example, a recent study exploring school-based occupational therapy practice in Ireland revealed that the most frequent reason for referral to pediatric occupational therapy was SI-based (94%) and that “sensory interventions” were the most frequently used (88.6%) (O’Donoghue et al., 2021). Further, despite some proponents of ASI® noting that the underpinning principles of neuroplasticity are linked with specific characteristics of pediatric populations (Smith Roley et al., 2007), SI-based occupational therapy is increasingly used with non-paediatric populations (e.g., mental health [Scanlan & Novak, 2015]; dementia and Alzheimers [Smith & D’Amico, 2020] and more).

Upton et al. (2014) call for further research to explore occupational therapists’ understanding of EBP. In relation to SI-based practice, Barton et al. (2015) reinforce this call, recommending that “future research should examine factors that impact treatment decisions” (p. 78). Terminological confusion (Reynolds et al., 2017), challenges in identifying intervention studies which fully adhere to ASI® fidelity (Pfeiffer et al., 2018), use of SI-based interventions across a range of clinical populations, and the fact that some clinicians may not be attuned to the complexities of the theoretical debate, must be assumed to impact on occupational therapists’ understanding of sensory interventions (Brown et al., 2019) with impacts on translation of SI-based evidence into clinical practice.

As noted, the more objective components of EBP are integrated through clinical expertise, which is subjective and dependent on an individual's ability to use knowledge effectively. In light of the arguable evidence-related contention surrounding SI-based occupational therapy, these interventions may offer a unique opportunity to investigate the subjective, individual nature of the clinical expertise component of EBP in a widely used, yet arguably contentious, arena of clinical occupational therapy practice. Intended to shed light on subjective, individual factors that are key to the real-world application of research evidence, this explorative, qualitative study interrogated occupational therapists’ motivation to offer SI-based interventions. Specifically, this study aimed to address the following research question: “What factors inform OTs’ use of sensory integration-based interventions within their clinical decision making, in the face of an academically-debated research evidence-base?”

## Methods

### Study Design

Data analysis was based in an interpretivist epistemology (Scotland 2012) following the ontological position of relativism (Guba & Lincoln, 1994). Qualitative interviews were employed as this aligns with the ontological position that recognises multiple truths (Sale et al., 2002).

### Participants

In the United Kingdom, ASI® and SI-based interventions are predominantly practiced by registered occupational therapists. Inclusion criteria stipulated: Health and Care Professions Council registration (in the UK, occupational therapy graduates can only be registered to practice after successful completion of a pre-registration Bachelors or Masters programme, so it can be assumed that all participants had [at minimum] a pre-registration BSc degree), practicing occupational therapists and completion of postgraduate SI training (up to SI Network [UK and Ireland] level 2 [in-depth understanding of key concepts, models and patterns of SI difficulty and assessment principles] or 3 [fully-qualified to carry out intervention according to ASI® fidelity process elements {Parham et al., 2011}]). These criteria were selected to ensure that respondents had sufficient knowledge and experience to maximize the relevance to the study objectives. All participants (*n* = 6) had completed accredited SI training after five or more years of clinical occupational therapy practice and self-reported to regularly use their SI training in clinical practice. To address growing tendencies to apply SI principles across varied populations (May-Benson & Kinnealey, 2012), occupational therapists were included regardless of the population with whom they work. Table 1 describes participant characteristics.

**Table 1:** Participant Details.

Participant	Years of occupational therapy experience (experience since completing SI 2/3 training)	Clients	Sector
1	9 (1)	Adults: Learning Disabilities	NHS
2	9 (4)	Children: ASD	Private
3	13 (3)	Children: ASD and Learning Disabilities (previously adult learning disabilities)	Private
4	10 (3)	Children: ASD	Private
5	13 (1)	Adults: Learning Disabilities	NHS
6	21 (1)	Children: ASD and Learning Disabilities (previously adult learning disabilities)	Private

### Data Collection

This study received ethical approval from Coventry University (#P36523). Purposive sampling was used for recruitment, via gatekeeper contact through SI organizations in the UK (e.g., SI Network UK & Ireland, and online SI discussion fora). Gatekeepers were provided with detailed participant information sheets detailing the study purpose, nature of participant involvement and commitment to confidentiality and anonymity, and were asked to distribute these to potential participants. These sheets were distributed via newsletters to all members of the organisations and networks, clarified participant rights to withdrawal, as well as processes for secure, encrypted and anonymised data storage. Within the ethics application, a window of time was indicated for recruitment, and a minimum sample size of between 4 and 6 participants was indicated. All participants who expressed interest in participating (during the recruitment window period; *n* = 6) were eligible and all provided informed consent.

A pilot interview was conducted with the first respondent to ensure that the semi-structured interview questions elicited depth of data with relevance to the study objectives; this participant was asked to provide feedback on the interview process. No adjustments were made to the interview protocol and data from the pilot participant was included in the final analysis. Semi-structured, individual interviews (∼45 min) were conducted by the first author (Rihtman, Tanya), either face-to-face (*n* = 4) or via Skype (*n* = 2). Interviews comprising two key questions with prompts (Appendix 1), were audio-recorded and transcribed verbatim. After transcription, participants were given the opportunity for member-checking, resulting in additions to one transcript.

In relation to author positionality, a pediatric occupational therapist (Bird, Diane) proposed the study when she was a pre-registration occupational therapy student out of curiosity as to why occupational therapists were using a clinical approach with a debated evidence base. The second author (Rihtman, Tanya) had more than 15 years of pediatric clinical and research experience, was trained in SI (UK level 3/international SI accreditation), and was mindful of the fact that the clinical application of evidence in the translation of evidence into practice is susceptible to subjective, individual factors that have not been sufficiently explored.

### Data Analysis

Transcribed interviews amounted to a data set of over 40,000 words; adherence to the five phases of thematic coding analysis (Table 2) (Robson & McCartan, 2016, p. 460–478) ensured a principled, systematic means of summarizing key features of the data.

**Table 2:** Application of the Five Phases of Thematic Coding Analysis.

Phase	Description
1. Familiarisation with data	The first author (DB) transcribed all interviews. The second author (TR) reviewed all the anonymised transcripts to ensure that both researchers were thoroughly familiar with all the raw data.
2. Generating initial codes	Robson and McCartan (2016, p. 468–469) emphasise that the flexibility of thematic coding analysis allows for the approach to be based on either inductive or deductive reasoning, or both. A pragmatic mix of both was used; inductive reasoning supported the identification of codes and themes that arose from the data, while the research objectives and knowledge of sensory integration-based literature which informed that coding process contributed an element of deductive reasoning. This process was undertaken by the first author (DB) and verified by the second author (TR). “Codes” were a word or short phrase that represented either the semantic or latent content of the chunk of text to which they were assigned. A systematic process, as recommended for thematic coding analysis (2016, p. 467–468) was used. Over 100 unique initial codes were generated.
3. Identifying Themes & 4. Constructing Thematic Networks	Initial “categories”, each with several associated codes, were identified and represented by highlighting associated extracts in different colours. All of the data extracts relevant to each code, from across the entire data set, were then grouped together under their associated categories. Each data extract was prefaced with both the transcript and line numbers to allow verification of each extract in its original context, and contributing to an audit-trail. This process was undertaken by the first author (DB) and verified by the second author (TR). This process was undertaken in an iterative manner such that no discrepancies arose in relation to verification of categories. The categories and their associated codes were then summarised and a detailed thematic map was hand-drawn by both authors to show relationships between all categories and codes. This enabled visualisation of the categories which could appropriately be combined to form themes and subthemes, as well as the relationships between themes and subthemes. The thematic network presented in Figure 1 illustrates inter-relationships between themes.
5. Integration and Interpretation	See “Results and Discussion.”

## Results and Discussion

**Figure 1.**
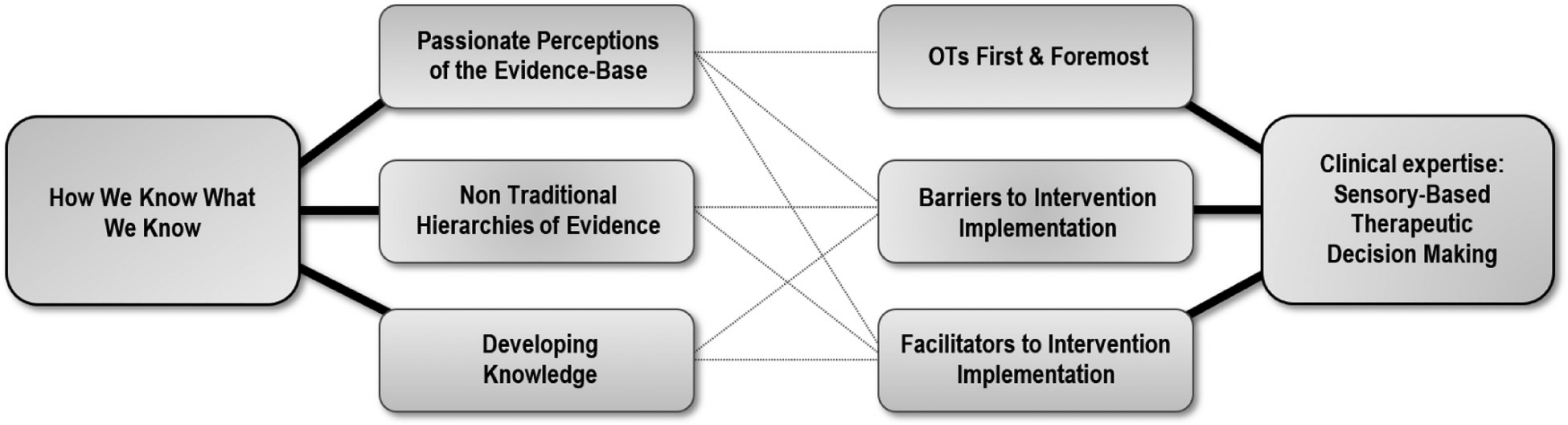
Thematic network.

Following the five phases of thematic coding analysis (Robson & McCartan, 2016), over 100 unique initial codes were generated (phase 2), which were then organised into categories with associated codes (phase 3) and summarised into a thematic network (phase 4) (Figure 1). Two main themes were identified, each with three subthemes, as well as bi-directional relationships between subthemes.

### Theme 1: How We Know What We Know

All participants discussed drawing upon various sources and types of information to inform their clinical decision making, including their own clinical experience, which they perceived to form part of the evidence base influencing their SI-based practice.

#### Subtheme 1.1: Passionate Perceptions of the Evidence Base

Participants’ perceptions over the strength of the evidence base varied; for example, those working with adults with learning disabilities acknowledged that less research on sensory-based integration therapy with this population has been conducted. There was a general perception that the sensory evidence base is growing stronger (“*sensory integration therapy…has a good evidence-base, and it is small, very, very small, but it is growing*” [P3]), and an RCT published by Schaaf et al., in 2014 was perceived as a significant development. Some participants referred to the claim that ASI now meets standards for EBP according to the criteria of the Council for Exceptional Children (Schoen et al., 2019), with P1 stating that “*Ayres SI is now classed as EBP.*”

The development of the Data Driven Decision Making tool (Schaaf, 2015), the ASI-FM criteria (Parham et al., 2011) and plans to develop the Evaluation in Ayres Sensory Integration® assessment (Mailloux et al., 2018) were perceived as means for strengthening the robustness of current and future studies, and the sense that “*all these kind of process elements are really…helping to strengthen our case”*[P3]. The emotive nature of this phrasing (*“our case”*) highlights the passionate perceptions held by participants to defend the use of these approaches in their clinical practice.

Some participants spoke of the personal responsibility of occupational therapists to contribute to the sensory knowledge-base, as well as the feeling that the UK lags in this area of research (“*it's not in the UK, we’re not good at all about research and evidence, it is coming from the States and from Canada and a little bit from Australia*” [P3]). It was felt that UK occupational therapists need to get better at publishing SI-based research (“*We should write more articles because actually when you get the time there are so many good things going on isn’t there*” [P6]) with one participant stating, “*I look at some of the outcomes I’ve had from my clients, and I think, you know, we’ve had great results, but it is about showcasing that and evidencing it [P1].*”

These participants appear to be highlighting the importance of individual practitioners’ evidence contributions, reflecting an acceptance of evidence across varying levels of traditional hierarchies of evidence (Tomlin & Borgetto, 2011) and acknowledging the potential contribution of different well-structured (Tomlin & Borgetto, 2011) methodological research designs to the sensory knowledge-base. Moreover, this theme suggests the centrality of personal therapeutic experience in contributing to strength of feeling about types of evidence underpinning “clinical expertise,” and aligns with the premise of the “research pyramid…[which] is the first attempt…to resolve the evidence tangle and provide a model that treats all evidence important for OT practice equitably” (Tomlin & Borgetto, 2011, p. 190).

#### Subtheme 1.2: Non-Traditional Hierarchies of Evidence

Academic debates surrounding the SI evidence base did not cause participants to doubt their practice. They were all strongly motivated to implement SI-based therapy by what they had personally witnessed, with one participant describing a hierarchy of influences: “*At the top of this pyramid would be my mentors, the very, very experienced therapists that have done it for a very long time, then it would be experience of other colleagues that have similar work experience and training, and then it would be what I see for myself and what I do*” [P2]. This seems to align well with the analogy of clinicians as “architects of EBP” (Paez, 2018) which suggests that expert opinion should not be viewed as the lowest form of evidence, but rather a parallel source of knowledge that complements EBP.

Modern definitions of EBP recognise the contribution of professional expertise and the client's preferences, alongside research evidence (Gerrish, 2010, p. 488). As explained by P2, “*from my day to day practice, I can see how children respond to this frame of reference…, so this is good enough for me as evidence.*” This tendency, however, needs to be viewed with extreme caution; while understanding how these “non-traditional hierarchies of evidence” are used by occupational therapists, Lee and Hunsley (2015) note that, although dramatic changes in a client's functioning may appear to provide compelling evidence of a therapy's impact, practitioners need to be scientific in their thinking because unsystematic clinical observations can lead to erroneous conclusions about treatment efficacy.

On the other hand, evidence hierarchies which locate measurable, inferential data as more important than qualitative evidence are increasingly being questioned. An OT Research Pyramid has been suggested (Tomlin & Borgetto, 2011), designed around a more equitable weighting of different forms of knowing, while simultaneously incorporating all forms of occupational therapy clinical reasoning, from simple to complex (Unsworth, 2017). The unequivocal views of the current study participants point towards flexible clinical reasoning being vital to how clinical expertise is used in EBP. If procedural, interactive and conditional reasoning (Fleming, 1991) lie at the center of therapeutic relations, then it must be assumed that occupational therapists, by definition, are required to apply research evidence flexibly and in relation to various, context-specific questions.

#### Subtheme 1.3: Developing Knowledge

All participants showed a desire to keep abreast of sensory developments, however the sheer volume of new articles in this field explains why busy practitioners tend to rely on clinical networks for research updates. Sources of information included conferences, books, journals, trade magazines, peers and mentors, and were described as follows “*I’ve done a lot of courses recently…, (a conference) I went to where the people who trained with Jean Ayres were talking and there was a lot of cases presented in terms of individual research projects that people had done around the efficacy of SI with different client groups, so picking ideas and things out of those…, research articles whether those be singular case studies or you know the bigger research articles, and also support from the local SI network and sharing of practice and peer supervision again I’m working closely with other OTs that are practicing SI. So kind of a whole wide range of sources [P4].*”

Participants appeared to be gaining information about SI-based occupational therapy from knowledge sources that predominantly support their existing views (e.g., P6 described “sensory goggles” as enabling understanding from a “*completely different perspective*”). However, clinicians need to guard against only using sources that support their existing viewpoint and should seek out and interroge the evidence for opposing views; this risk of “sensory goggles” becoming “sensory blinkers” could be argued to be a risk for all occupational therapists, not only in relation to SI-based occupational therapy.

This subtheme serves as a reminder of the risks of subjective clinical expertise becoming a potential distractor to an objective understanding of research evidence. The importance of developing knowledge that is based on a wide variety of potentially opposing literature appears to reflect the advice of Pollock (2009, p. 9): “Remember the old adage ‘If the only tool you have is a hammer, you’ll view every problem as a nail’.” Further, this aligns with the importance of occupational therapists adopting regular structured reflection practice if quality EBP is to be achieved (Thomas & Law, 2013).

### Theme 2: Clinical Expertise: SI-Based Therapeutic Decision Making

As the leading professionals supporting people with sensory difficulties (Bodison & Parham, 2018), the fundamental ethos of the occupational therapy role appeared to underpin clinical choices, with SI being viewed as “*only a tool in the toolbox of OT*” [P4]. This aligns closely with the integration of clinical reasoning as part of a more fit-for-purpose way to view occupational therapy research evidence (Tomlin & Borgetto, 2011) as well as the need for a multifaceted, functional approach to using SI-based evidence (Reynolds et al., 2017). Moreover, this reflects the ability to “use clinical skills and past experience to rapidly identify each patient's unique [situation]” (Straus et al., 2018, p. 13).

#### Subtheme 2.1: Occupational Therapists First and Foremost

The relevance of sensory interventions to occupational therapy practice was highlighted by participants as an effective way to address clients’ occupational performance difficulties, however, one participant raised concern about potential external perceptions that “*OTs have almost moved away from OT and it's all sensory, sensory, sensory*” [P1]. This external perception is at odds with participants’ self-perception, who described themselves as “*OTs first and foremost*” [P1]. Indeed, the American Occupational Therapy Association (AOTA) SI fact sheet (AOTA, n.d.) appears to share participants’ views of both effectiveness and relevance of SI to occupation, stating that these “interventions promote occupational performance and help individuals maximize participation in daily living activities.” As P3 put it, “*I’m an OT and I am focussed on the occupation and the function first and foremost, and I just find that sensory integration is a fantastic way of helping some of the children and adults to gain a greater control over their everyday activities.*” The idea of being occupational therapists first and foremost presupposes a holistic and functional worldview and suggests that occupational therapists who use SI-based interventions are leaving the nuances of the sensory debate to academia, and are applying the evidence flexibly when faced with real world, functional needs.

From this functional perspective, participants highlighted the potential of sensory strategies to make a difference to clients’ lives: “*I look at the people I work with and… families, that have just said to me, ‘You’ve changed our lives, you’ve made a massive difference’*” [P5]. Making a difference has been found to be a significant source of occupational meaning and purpose for occupational therapists (Smith & Kinsella, 2009). As therapists in context (Pentland et al., 2018), occupational therapists’ behaviour and actions are informed by environmental contexts and resources as well as their emotions. The truly meaningful moments for an occupational therapy may not always be easily articulated, but a participant described “*that magic moment when a child becomes modulated…when you know you are doing the right thing*” [P2]. This professional meaning and purpose that sensory interventions may facilitate was highlighted by one participant who stated that “*it can make such a massive difference to people's lives and I’ve seen it. I’ve seen what happens and that is why I am so passionate about it*” [P6]*.* It is possible, then, that in addition to the suitability to the clients’ needs, sensory interventions are selected from the occupational therapy toolkit due to practitioners’ optimism (Cane et al., 2012) and belief that it works and thus has potential to facilitate professional meaning for the practitioner, because “*to see those light bulb moments is amazing*” [P4].

This is closely linked with the subtheme of passionate perceptions of the evidence base. The differences in how the efficacy and value of sensory interventions are perceived and represented within different factions of the occupational therapy community may be underpinned by philosophical differences over whether this largely bottom-up approach fits within a profession that claims occupation as its core tenet. Yet some participants suggested that sensory interventions are foundational and are necessary before their clients can benefit from top-down approaches. “*Sometimes if you go in top down you can resolve an issue much quicker and much swifter, and you would do that. But…, I find this with kids with learning disabilities, if I want to get them to sit down and be able to do a matching activity I can’t get them to sit down let alone do the matching activity,… what I’m interested in is trying to get them regulated through the day….it's that underlying skills of regulation…which I think SI is just absolutely fabulous for because if you can get that right then it opens up a whole load of opportunities for the child or the adult…. when you’ve got them regulated with the bottom up you can start teaching them some things in a top down approach.*” [P6]

This subtheme additionally highlights the embeddedness of person-centered care within the patient preferences component of EBP (Haynes et al., 2002). Service-users are requesting SI-based occupational therapy interventions to address the characteristics of their clinical circumstances. Existing models of EBP place equal weighting on clinical expertise, patient preference and research evidence (Haynes et al., 2002). If the focus on research evidence overshadows the focus on the other components of EBP, true adherence to EBP could be called into question. While those who debate SI-based interventions from a theoretical perspective may disagree, it could be argued that the findings of this study demonstrate that in the “real world” of clinical practice, occupational therapists are indeed using their clinical expertise to incorporate patient preferences and clinical state and circumstances in the service of supporting functional intervention outcomes.

#### Subtheme 2.2: Barriers to Intervention Implementation

SI-based clinical choices are impacted by practical limitations inherent in the approach itself, reinforcing the fact that clinical expertise is founded on the need for pragmatic application of available research evidence. For example, difficulties in accessing treatment rooms with suspended equipment (“*because not everybody can come to a super-duper room with swings*” [P3]) may result in difficulty in meeting the ASI-FM criterion of “presenting sensory opportunities” (Parham et al., 2011) (“*there is debate about whether you can say that you are doing SI without suspended equipment*” [P4]). Limitations of adherence to the ASI-FM appeared to be particularly relevant within UK National Health Service (NHS) contexts. Additionally, an apparent lack of SI commissioner knowledge (“*commissioners…are not going to know that there is a robust method*” [P1]) may form barriers for implementation of SI-based occupational therapy. As P5 highlighted, “*because of the fidelity tool, I feel very uncomfortable with…trying to…maintain it [ASI practice] and I can’t …do that in the time that I’ve got, the responsibilities that I’ve got.*” This does not preclude private practitioners from struggling to adhere to ASI-FM guidelines; “*I don’t think it is very practical from my experience to try to meet those criteria of the fidelity tool on every single session that you have. …it's certainly a very good guide to treatment but I wouldn’t say that I fill in every area on every single Ayres session that I have*” [P2]. These structural barriers may be preventing therapists from implementing a clearly articulated therapeutic approach (be it ASI® or SI-based) based on available research evidence.

Creating and disseminating knowledge is insufficient to ensure its effective use in decision making, and “sound application of knowledge to improve health” (Straus et al., 2009, p. 165) is viewed as the key to knowledge translation. This study highlights the importance of those working in research being mindful that its application via the clinical expertise of the practitioner—that is, knowledge translation—will always occur within the context of pragmatic and potentially economic realities and in response to patient preferences. In the case of SI-based occupational therapy, “*a lot of parents already know that is what they want for their child and they’ve done a lot, a lot of research around what they want and it's almost kind of spurred them more on to try and get it for their children*” [P3], which may lead those with the financial means to seek these interventions privately. The ability of the occupational therapy to incorporate patient preferences as part of their clinical expertise may then be claimed to be economically driven, with resultant ethical clinical reasoning impacts. Further, despite the equal weighting of patient preferences within EBP, the clinician cannot “escape the laws of science or nature, despite the clients’ preferences. The clinician cannot ethically prescribe [potentially] ineffective … interventions solely to please … patients” (Paez, 2018, p. 223).

Barriers to making sensory choices are closely linked with all subthemes of “How We Know What We Know.” The decisions and behaviours of the occupational therapy are likely to be informed by a complex interplay of various behavioural, knowledge and skill-based constructs, and their expression for the individual practitioner (Cane et al., 2012). Having to sift through large volumes of information, argue for non-traditional forms of evidence and navigate controversies surrounding this area of clinical occupational therapy practice may form a barrier to selecting sensory interventions.

#### Subtheme 2.3: Facilitators to Intervention Implementation

Despite the above-mentioned barriers, participants noted key facilitators supporting their decisions to offer SI-based occupational therapy, as well as the potential positive links with the three subthemes of theme 1. Foremost, their decisions were supported by their clients having sensory processing difficulties (i.e., clinical state and circumstances) and their firmly held belief that sensory approaches are effective: “*taking a sensory approach and then educating everyone around it can make such a massive difference to people's lives and I’ve seen it. I’ve seen what happens, that is why I am so passionate about it*” [P6]. This belief is closely linked with the themes of non-traditional hierarchies of evidence and developing knowledge. The currently expanding range of evidence (Pfeiffer et al., 2018) gives occupational therapists potential for accessing information and support for their decisions that they would not previously have had.

Most participants talked about how other occupational therapy interventions had not been effective with some of their clients, and that sensory approaches addressed this gap. “*I’d practiced in pediatrics for a number of years and felt like there was something missing. I felt that I was practicing and putting treatment programs into place and they weren’t working and … once I’d then gone and done my (SI) training…I could see why they weren’t working. It was the missing part of the jigsaw*” [P4]. Likewise, P2 indicated that they sought SI training from the need to “*find something that is more holistic, more integral, more comprehensive as an approach to therapy.*”

In this regard, there was particular appreciation of how SI theory enabled participants to both better understand their clients themselves as well as better articulate the clients’ difficulties and behaviors to families and other professionals. Occupational therapists are facilitated by their own experiences in this regard, they trust what they have personally witnessed, and thus hold strong views. One participant succinctly expressed “*I have seen it to be effective, therefore I believe it to be effective*” [P3]. Aligning closely with beliefs about consequences, specifically outcome expectancies (Cane et al., 2012), it could be argued that this is central to what is intended by “clinical expertise.”

Finally, recent increases in availability of clinical tools that formalize occupational therapy sensory interventions also support practitioners in choosing these interventions. The ASI-FM helps support and structure SI practice (“*actually having that fidelity tool has helped in guiding my thinking and thought processes*” [P4]), and plays a role in protecting the art of SI within the profession, as well as contributing to research (“*I really like the fact that it has all been formalized because that really helps from a research perspective and it really helps for us to be able to know what is [A]SI and what's not [A]SI*” [P6]). As P4 noted, “*within the fidelity tool there are things around training and supervision and support so…people can’t pick up a sensory profile and say that they are doing SI and say that they are trained therapists…*”

### Limitations

This study was limited to six participants, which potentially limits the diversity of views uncovered by the study. However, having participants from different employment sectors and different client groups facilitated the contribution of a range of perspectives to the study. In the UK, occupational therapy use of ASI® conforming to the ASI-FM criteria is rare. Although the participants were familiar with the ASI-FM criteria, the realities of their practice require flexibility in its application, such that this could potentially impact on their clinical application of available research evidence, which may in turn impact on the exploration of clinical expertise when implementing SI-based occupational therapy. Exploratory qualitative studies are at risk of researcher bias, as those proposing the study can be assumed to have particular interest in the topic which might shape the direction of the study. This was addressed as far as possible by identifying and reflecting on researcher positionality, and adhering to a clear process of analysis which included verification by one author of the themes identified by the other author, and the maintenance of a reflexive research diary throughout the study to ensure elimination of any potential researcher bias remained at the forefront of the process. Finally, although the TDF has been a useful lens through which to consider some of the study findings (Cane et al., 2012), the initial design of the study in line with this framework (e.g., structuring the interview guide in line with the different TDF domains) may have been a useful approach to adopt.

## Conclusions and Future Directions

The current study interrogated the subjective, individual factor of clinical expertise as the mechanism through practitioners implements the other components of EBP, within an area of clinical practice with an academically-debated research evidence base. It is important to emphasize that the aim of this study was not to evaluate the interventions themselves, nor to evaluate the strength of the evidence to either side of any academic debate, but to understand why occupational therapists working in a realm with a myriad of strong opinions adopt the clinical practices that they do in the translation of research knowledge. By exploring the views of occupational therapists who practice using these approaches, it has been possible to offer a snapshot into how occupational therapists use their clinical expertise to equally—yet flexibly—consider all three components of EBP. Occupational therapists appear to be using these approaches in a flexible, person-centered way to support the attainment of functional, occupational outcomes, adapting pragmatically within the “real world” of clinical practice. However, the sources of information that occupational therapists access may introduce an inherent bias and reinforce the importance of structured reflective practice if this potential bias in clinical decision making is to be addressed.

The process of knowledge creation is always developing and available evidence is always incomplete. Moreover, faced with the same information, people may select different studies to consider, consequently coming to differing conclusions, reinforcing the importance of adopting a clear theoretical framework such as the TDF to support the interpretation of factors informing the specific processes underlying successful behaviour change (Cane et al., 2012). The study findings have potential to shed light on the subjective, individual factors of the clinical expertise component of EBP when research knowledge is translated into clinical practice and raise important directions for future research related to research and EBP behaviour change of occupational therapists. For example, future studies examining occupational therapy EBP and knowledge translation might explore the role of each domain of the TDF with specific application to the occupational therapy profession, or use the TDF to explore reasons underlying occupational therapy-specific research implementation challenges and behaviour change. Finally, findings reinforce the importance of clinicians having the skills to critically appraise all forms of information that inform their practice so that evidence can be applied not only based on “what works,” but “what works for who, when, and where.”

## Key Messages

• Research application is inherently subjective, as clinical practice realities can never imitate the carefully controlled conditions of empirical research.
• Authentic knowledge translation can only occur if subjective, individual factors of clinical decision making are better understood in relation to applying controlled empirical evidence in uncontrolled clinical realities.
• Clinically practising occupational therapists are committed to evidence-based practice, and appear to use complex clinical reasoning flexibly across a range of hierarchies of evidence when translating research into practice.

## Appendix

### Appendix 1: Semi-Structured Interview Guide

Question 1: Why do you practice sensory integration based therapy?

Examples of follow-up or prompt questions:

- What originally motivated you to become an SI practitioner?
- What factors have influenced your choice to offer sensory integration-based therapies?
- Do you have anything else you would like to tell me about why you practice sensory integration-based therapy?

Question 2: What are your thoughts about the current evidence base for SI based practice?

Examples of follow-up or prompt questions:

- What types of evidence do you use to support your practice?
- What are the sources of the evidence that you use?
- How would you describe the relative usefulness of research evidence, your own clinical experiences, and expert opinion to your sensory integration-based practice?
- Can you tell me your thoughts about the evidence base for the efficacy of sensory integration-based therapies with your client group?
- Do differing schools of thought over the strength of the evidence base for sensory integration-based therapies affect your practice? If so, how?
- Do you have anything else you would like to tell me about your own, or others, views of the evidence base for sensory integration-based therapy?
